# Large-Scale Preparation of Highly Stable Recombinant Human Acidic Fibroblast Growth Factor in *Escherichia coli* BL21(DE3) plysS Strain

**DOI:** 10.3389/fbioe.2021.641505

**Published:** 2021-04-13

**Authors:** Bingjie Yu, Wenzhe Sun, Zhen Huang, Gang Sun, Le Li, Jiawei Gu, Mengying Zheng, Xiaokun Li, ChangJu Chun, Qi Hui, Xiaojie Wang

**Affiliations:** ^1^Wenzhou Medical University, Chashan University Park, Wenzhou, China; ^2^College of Pharmacy, Chonnam National University, Gwangju, South Korea; ^3^Engineering Laboratory of Zhejiang Province for Pharmaceutical Development of Growth Factors, Biomedical Collaborative Innovation Center of Wenzhou, Wenzhou, China; ^4^Research Institute of Pharmaceutical Sciences, College of Pharmacy, Chonnam National University, Gwangju, South Korea

**Keywords:** rhaFGF_135_, *Escherichia coli* BL21(DE3)plysS, fermentation, purification, wound healing

## Abstract

In this study, the optimum human *aFGF* gene encoding haFGF_135_ was cloned in pET3c and transferred to *Escherichia coli* BL21(DE3) plysS. To enhance the yield of fermentation and the expression level of the target protein, the fermentation parameters, including temperature, pH, dissolved oxygen, glucose concentration, ammonium chloride concentration, induction time, and inducer (IPTG) concentration, were optimized. The optimized fermentation parameters were used in large-scale fermentation (30 L). Ion-exchange and heparin-affinity column chromatography techniques were used for separation and purification of rhaFGF_135_ protein. HPLC, isoelectric focusing electrophoresis, and mass spectrometry were used to detect the purity, isoelectric point, and molecular weight and peptide map of rhaFGF_135_ protein, respectively. Mitogenic activity of rhaFGF_135_ protein was detected in NIH-3T3 cells and a full-thickness injury wound diabetic rat model. The production and expression level of rhaFGF_135_ in the 30-L scale fermentation reached 80.4 ± 2.7 g/L culture and 37.8% ± 1.8%, respectively. The RP-HPLC and SDS-PAGE purity of the final rhaFGF_135_ product almost reached 100%, and the final pure protein yield was 158.6 ± 6.8 mg/L culture. Finally, the cell and animal experiments showed that rhaFGF_135_ retained a potent mitogenic activity. The large-scale process of rhaFGF_135_ production reported herein is relatively stable and time-saving, and thus, it can be used as an efficient and economic strategy for the synthesis of rhaFGF_135_ at the industrial level.

## Introduction

Human acidic fibroblast growth factor (haFGF), also called FGF1, is a single-chain heparin-binding protein from the fibroblast growth factor (FGF) family (Burgess, 1991). It is a broad-spectrum mitogen, a powerful vasodilator, and a potent neuromodulator that can strongly bind to all four known FGF receptors. The development of recombinant DNA technology has enabled researchers to successfully express haFGF in various hosts, including *Escherichia coli*, yeast, silkworm, and mammalian cells (Wu et al., 2005; Fantoni et al., 2007; Zhou et al., 2011; Wang et al., 2015). Of these hosts, *E. coli* is ideal for the production of recombinant proteins on an industrial level (Yin et al., 2007) due to its unique advantages, including ease of genetic manipulation, inexpensive culture medium, and fast protein expression (Oberg et al., 1994; Wu et al., 2001; Chen, 2012; Papaneophytou and Kontopidis, 2014). In fact, Wu et al. and Wang et al. have succeeded in expressing haFGF in *E. coli* at the scales of 3 and 20-L, respectively (Wu et al., 2005; Wang S. M. et al., 2009). In addition to DNA recombination, high-density cell fermentation technology has developed rapidly in recent years. Today, this technology is considered one of the most effective methods of recombinant protein production.

The native human aFGF is composed of 154 amino acids, including 19 *N*-terminal amino acids that are analogous to those found in human interleukin-1 (IL-1). This indicates that haFGF may produce the same endogenous immunoresponse as IL-1 and that the two proteins have similar biological functions, such as activation of macrophages, cell modulation, and growth arrest (Venkataraman et al., 1999). Furthermore, the structural scaffold of proinflammatory cytokine IL-1 is identical to that of aFGF/bFGF; thus, the two compete for the same receptor binding sites in tyrosine kinase domains (Minter et al., 1996).

In this study, we established a shortened version of human aFGF (aFGF_135_), comprising 135 amino acids, with a theoretical molecular weight of 15.4 kDa. To construct this protein, 20 of the original 154 amino acids were deleted from the *N*-terminal, and 1 alanine (Ala) residue was added just before the terminal. It was previously shown that such a protein exhibited improved stability (Cheng and Kuo, 2011) and a non-significant difference in biological activity (Yang et al., 2015). A high cell-density fermentation process was also developed herein for large-scale production of aFGF_135_ in a 30-L fermenter, and the optimal fermentation conditions were determined.

## Materials and Methods

### Materials

Tryptone and yeast powder were purchased from OXOID Co., Ltd. (Hampshire, England). The NIH-3T3 cell line and pET3c plasmids were supplied by ATCC and BGI, respectively. Restriction enzymes, gel extraction kit, PCR purification kit, and plasmid micro-preparation kit were obtained from Dalian Takara Corporation (Dalian, China). Isopropyl-β-D-thiogalactoside (IPTG), ampicillin sodium, and 30-L fermenter were purchased from Beijing Dingguo Changsheng Biotechnology Co., Ltd., CSPC Zhongnuo Pharmaceutical (Shijiazhuang) Co., Ltd., and Shanghai Baoxing Bio-Engineering Equipment Co., Ltd., respectively. CM-Sepharose and heparin-Sepharose were provided by GE Healthcare (United States). Polyclonal monkey anti-human aFGF antibody and *E. coli* BL21(DE3) plysS (Catalog No. CD601) were obtained from Santa Cruz Biotechnology (United States) and Transgen Biotechnology Co., Ltd. (Beijing, China), respectively. The human FGF1 standard was identified by Shanghai Institutes for Biological Sciences at the Chinese Academy of Sciences.

### Construction and Identification of the pET3c/rhaFGF_135_ Expression Vector

An optimized gene sequence of 408 bp encoding haFGF_135_ (GenBank accession number MT150274) was designed based on the human FGF1 cDNA data retrieved from the NCBI database (reference sequence: NM_001144892.2, base number: 73-540). The developed upstream and downstream primers (upstream, 5′-TTA ACT TTA AGA AGG AGA TAT ACA TAT GGC TAA CTA TAA AAA ACC-3′; downstream, 5′-CTT TCG GGC TTT GTT AGC AGC CGG ATC CTT AGT CCG ACG ACA C-3′) comprised *Nde*I and *Bam*HI sites, respectively. The optimized *haFGF*_135_ gene was synthesized by BGI Tech Solutions (Beijing Liu He) Co., Ltd. (Beijing, China) and amplified by polymerase chain reaction (PCR) (conditions: 94°C for 3 min; 30 cycles at 94°C for 30 s, 59°C for 30 s; 72°C for 42 s; a final extension at 72°C for 5 min). The amplified fragments were cut by the *Nde*I and *Bam*HI restriction enzymes at 37°C for 3 h and ligated with the pET3c vector at 16°C overnight, which had been previously digested with *Nde*I and *Bam*HI. The recombinant plasmid of pET3c/rhaFGF_135_ was sequenced by BGI Tech Solutions (Beijing Liu He) Co., Ltd., further confirmed by restriction enzyme analysis, and then transferred into the *E. coli* BL21(DE3) plysS strain.

### Induction and Expression of rhaFGF_135_

The induction of rhaFGF_135_ was performed as follows. The positive colonies were cultured in 5 mL LB sterile medium containing 100 μg/mL ampicillin sodium at 37°C and 200 rpm, and then 1 mM IPTG was added when the OD_600_ reached 0.8–1.2. After incubation for another 4 h at 37°C and 200 rpm, the expression level of rhaFGF_135_ was detected and estimated by 12% SDS-PAGE. Coomassie blue staining and densitometry were analyzed with Image Lab software. Western blotting analysis was used to identify rhaFGF_135_. The colony with the highest expression level of rhaFGF was taken as the seed strain in subsequent optimization of fermentation parameters or high-density fermentation.

### Optimization of the Fermentation Parameters for rhaFGF_135_

Temperature, pH, dissolved oxygen (DO), and other fermentation parameters affect the growth and expression of rhaFGF engineering strain. In order to obtain the best fermentation parameters, we did a screening experiment in a 250 mL shaking flask. The influencing factors and level design are shown in Table 1. According to the preliminary experiment, the volume of the medium below 30 mL was approximately equivalent to 30% oxygen concentration. Therefore, four medium volumes of 30, 50, 70, and 100 mL were designed to simulate the effects of oxygen concentrations on the growth curve and expression level of engineered strain. The shaking flask experiments were performed in triplicate as follows: the monoclonal engineered strain of rhaFGF_135_ was added to 30 mL LB medium containing 100 μg/mL ampicillin sodium and incubated at 37°C with 150 rpm. After incubation for 10 h, the seed culture was transferred into 30 mL fresh LB medium at a ratio of 1 to 100 (v/v) and incubated at 37°C with 200 rpm. When OD_600_ reached 0.8–1.2, 0.8 mM IPTG was added and induced for 4 h at 37°C with 200 rpm. The growth curves of the rhaFGF_135_ engineered strain with culture time were drawn at different temperature, pH, concentration of glucose, NH_4_Cl, and dissolved oxygen. Expression of rhaFGF_135_ with induction time was drawn at different concentrations of IPTG, glucose, NH_4_Cl, and DO, pH, and temperature. The expression level of rhaFGF_135_ was detected by 12% SDS-PAGE, Coomassie blue staining, and densitometry analysis with Image Lab software. The optimized fermentation parameters were then validated at the 2-L scale flask containing 300 mL LB medium fermentation.

**TABLE 1:** Optimization of the fermentation parameters for rhaFGF_135_.

Factors	Level						
	1	2	3	4	5	6	7
Temperature (°C)	30	33	35	37	/	/	/
Medium volume^a^ (mL)	30	50	75	100	/	/	/
NH_4_Cl (g/L)	0.5	1	2	4	10	/	/
pH	6.6	6.8	7.0	7.2	7.4	/	/
Glucose (g/L)	0.5	1	2	5	10	20	/
Induced OD_600_	0.2	0.4	0.8	1.2	1.8	2.5	/
Induced time (h)	0	1	2	3	4	5	6
IPTG (mM/L)	0.01	0.05	0.1	0.3	0.5	0.8	1

*^a^During optimization, after measuring by dissolved oxygen electrode, the dissolved oxygen ≥30%, when the volume of medium was 30 mL in 250-mL shake flask with 30 mL LB medium; the dissolved oxygen <30%, when the volume of medium was 50, 70, or 100 mL in 250-mL shake flask.*

### Large-Scale Fermentation Process of rhaFGF_135_

The seed strain of rhaFGF_135_ was activated in 30 mL LB medium containing 100 μg/mL ampicillin at 37°C and 200 rpm. When OD_600_ reached 0.8–1.2, the culture was added to 300 mL of the modified medium containing 10.0 g/L trytone, 10.0 g/L yeast extract, 4.0 g/L NaCl, 1.0 g/L KH_2_PO_4_, and 3.0 g/L K_2_HPO_4_. The mixture was then incubated at 37°C and 150 rpm for 10 to 12 h. Subsequently, the culture was transferred to a 12-L fermentation medium (1:10, v/v) consisting of 17.0 g/L tryptone, 23.0 g/L yeast extract, 4.0 g/L NaCl, 3.0 g/L K_2_HPO_4_, 1.0 g/L KH_2_PO_4_, 4.0 g/L NH_4_Cl, 5.0 g/L glucose, 0.6 g/L MgSO_4_, 13 mg/L CaCl_2_, and 5 mg/L vitamin B1. The new culture was incubated in a 30-L fermenter at 37°C. Within the first 2 h of incubation, 30% glucose solution was added at speed of 0.5 mL/min. Thereafter, the addition rate of glucose solution was adjusted based on the growth status of cells. When OD_600_ reached 22–25, 0.8 mM IPTG was added, induced for 1 h, and then the nitrogen source (17.0 g/L tryptone, 4.0 g/L NaCl, 23.0 g/L yeast extract, 3.0 g/L K_2_HPO_4_, 4.0 g/L MgSO_4_, and 1.0 g/L KH_2_PO_4_) was added. The protein expression level and cell density (OD_600_) in the culture medium were measured every hour. After 4 h of induction, the cells were collected and centrifuged with 16,000 rpm for 30 min at 4°C. The cell pellets were stored in a freezer at −80°C.

### Purification of rhaFGF_135_

All of the purification procedures were carried out at 4°C and monitored at 280 nm. The stored frozen cell pellets were thawed and resuspended in 20 mM ice-cold phosphate buffer (PB) (pH = 7.4) containing 0.1 mol/L NaCl, 5 mM/L EDTA-2Na, and 0.05% Tween-80 at a proportion of 1 g cell pellet per 10 mL PB buffer. After high-pressure homogenization, the mixture was centrifuged at 8,000 rpm for 40 min. Thereafter, the supernatant was collected and loaded onto a pre-equilibrated CM-Sepharose column (5.0 × 50 cm, 500 mL bed volume), followed by washing with 3 bed volumes of 20 mM PB (pH = 7.0, 5 mM EDTA-2Na, and 0.1 mol/L NaCl) until the baseline became stable. Proteins were eluted with 3–5 bed volumes of the buffer (20 mM/L, pH = 7.0, 5 mM EDTA-2Na, and 0.6 mol/L NaCl). After that, the pooled protein solution was loaded onto a heparin affinity column (3.5 × 60 cm, 250 mL bed volume) and washed with 3 bed volumes of PB (20 mM, pH = 7.0, 5 mM EDTA-2Na and 0.9 mol/L NaCl). Finally, the bound proteins were eluted with 1–2 bed volumes of the buffer (20 mM/L, pH = 7.0, 5 mM/L EDTA-2Na and 1.3 mol/L NaCl) and stored at −80°C. The concentration of aFGF_135_ was determined according to the Lowry method, and its purity was assessed using SDS-PAGE and RP-HPLC. The isoelectric point (pI) and biological activity of the protein were also examined. Western blotting, MALDI-TOF/MS, *N*-terminal sequencing, and molecular peptide mapping were used to evaluate the authenticity of the purified aFGF_135_.

### Bioactivity Assay of rhaFGF_135_

NIH-3T3 cells were cultured at 37°C and 5% CO_2_ in a DMEM low glucose medium (1.0 g/L glucose) containing 1% penicillin/streptomycin and 10% fetal bovine serum (FBS; Gibco; Thermo Fisher Scientific, Inc.). This cell culture was then transferred to a 96-well plate (7,000–9,000 cells/100 μL/well) and incubated for 24 h. Thereafter, the cells were serum (0.5% fetal bovine) starved for 18 to 24 h before replacing the culture medium with 120 μL of fresh DMEM low-sugar medium containing 0.5% FBS and 100 μg/mL heparin sodium. Subsequently, 40 μL of aFGF standard solution and rhaFGF_135_ stock solution was added to the cells, followed by four-fold gradient dilution. Each well was made in duplicate. After incubation for 48 h, 20 μL of MTT (5 mg/mL) was added to each well and incubated for another 4 h, and then the medium was replaced with 100 μL dimethyl sulfoxide (DMSO) in each well. Finally, the plate was oscillated for 5 min, and the absorbance measurements were recorded at 570 and 630 nm for signal and background readings, respectively.

### Animal Model and Experimental Groups

Male SD rats (180–220 g) received tail vein injection of STZ (70 mg/kg) dissolved in pH 4.5 citrate-citric acid buffer, within 30 min, once a day, for 2 days. Before the surgery, rats with high blood glucose levels (≥11.1 mmol/L) were selected for the full-thickness injury model. After general anesthesia with pentobarbital sodium at 45 mg/kg, the dorsal area was totally depilated. Subsequently, a full-thickness wound (1.8 cm in diameter) was made on one side of the rat’s back. After pressing the wound to stop bleeding, each rat was placed in a separate cage. In this model, rats were divided into two groups: control group and rhaFGF_135_ group (*n* = 7 per group). Control rats only received 0.2 mL physiological saline daily. The rhaFGF_135_ group rats were treated with rhaFGF_135_ solution at a dose of 90 AU/cm^2^ daily. The wound healing progress was observed daily and photographed at days 0, 7, 14, and 21 for calculating the wound area by Image Pro plus 6.0 software. The following equation was used to calculate the wound healing rate: *R*_*i*_ = (*A*_0_ − *A*_*i*_)/*A*_0_, where *A*_0_ is the wound area at 0 day and *A*_*i*_ is the wound area at each photographed day.

### Hematoxylin and Eosin Staining

At 21 days, rats from each group were randomly selected and the skin tissue around the wound was excised under deep anesthesia with 10% chloral hydrate (4.0 L/kg, i.p.) and fixed overnight in precooled 4% paraformaldehyde, followed by paraffin embedding. The transverse paraffin sections (5-μm thick) were placed on microscope slides for histopathological evaluation after hematoxylin and eosin (H&E) staining, which showed the general overview of fibroblasts, capillaries, and collagen fibers. Three rats per group were randomly selected for skin pathological evaluation via semiquantitative scoring from a specialist pathologist, as follows: no proliferation (0 points); mild proliferation (1 point); significant proliferation (2 points); and substantial proliferation (3 points).

### Statistical Methods and Analysis

All of the results presented herein were expressed as mean ± standard deviation. The statistical analysis of quantifiable results was performed using Student’s *t-*test with GraphPad Prism 5.0 software. The ordered categorical data were statistically analyzed by Ridit analysis. ^∗^*p* < 0.05, ^∗∗^*p* < 0.01, and ^∗∗∗^*p* < 0.001 signified statistically significant results.

## Results

### Plasmid Construction and Expression of rhaFGF_135_

**FIGURE 1:**
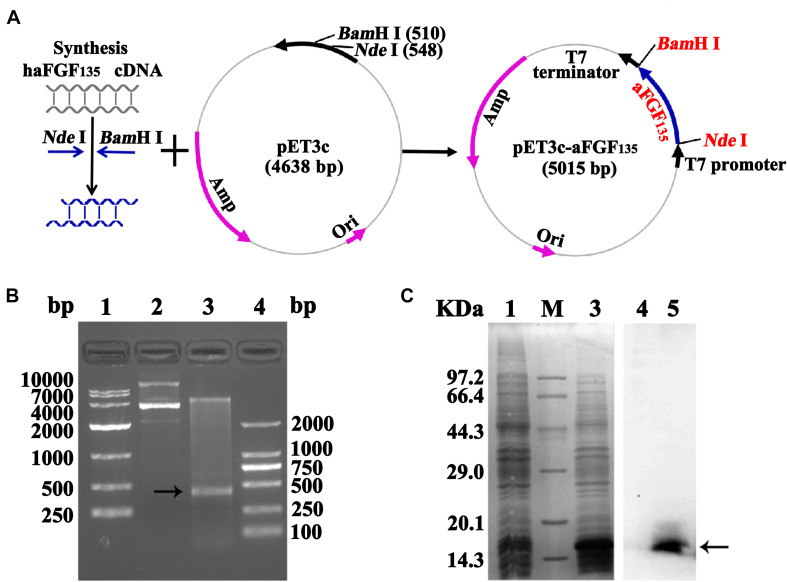
Recombinant plasmid construction and expression of the rhaFGF_135_ protein. **(A)** Schematic diagram of pET3c-rhaFGF_135_ recombinant plasmid construction. **(B)** Restriction enzyme analysis of the pET3c-rhaFGF_135_ recombinant plasmid. Lanes 1 and 4, DNA molecular weight marker. Lane 2, plasmid before digestion. Lane 3, plasmid after digestion. **(C)** SDS-PAGE (left) and western blot (right) analyses of the expressed rhaFGF_135_ protein in the lysis of *E. coli* BL21(DE3) plysS. Lanes 1 and 4, non-induced. Lanes 3 and 5, induced by IPTG for 4 h. Lane M, molecular weight marker. Black arrows indicate rhaFGF_135_ protein.

The construction process of human *aFGF*_135_ gene to pET3c vector is shown in Figure 1A. Restriction enzyme analysis was used to confirm whether *rhaFGF*_135_ gene was successfully linked with pET3c plasmid. As shown in Figure 1B (lane 3), there was a 420-bp fragment, indicating that rhaFGF_135_ gene was correctly integrated into the pET3c plasmid. Compared with pre-induction, a protein band was inducted at about 160 kDa, indicating that rhaFGF_135_ could be highly expressed (Figure 1C, lane 3). Additionally, western blotting indicated that rhaFGF_135_ protein was recognized by the human aFGF polyclonal antibody (Figure 1C, lane 5).

### Optimization of Fermentation Parameters

**FIGURE 2:**
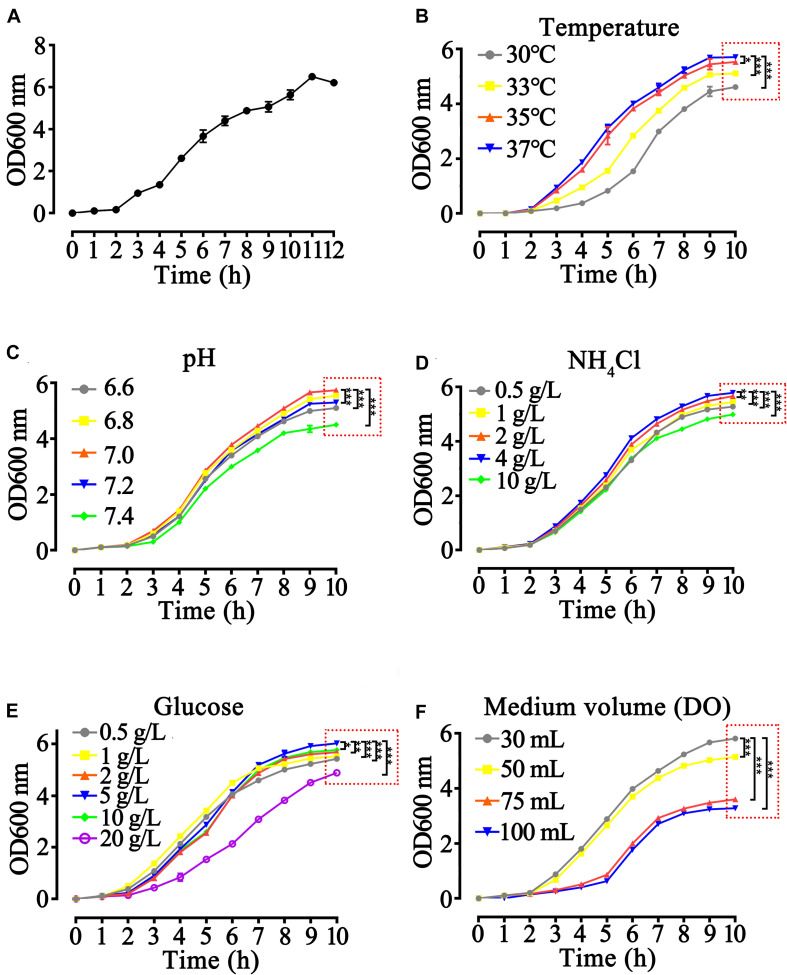
Optimization of growth parameters of rhaFGF_135_ *E. coli* strain in 30 mL LB medium. **(A)** The 12-hgrowth curve of the rhaFGF_135_ *E. coli* strain in 30 mL LB medium at 37°C, 200 rpm. **(B–F)** The 10-h growth curve of rhaFGF_135_ *E. coli* strain under different conditions, including **(B)** temperature of 30, 33, 35, and 37°C, **(C)** pH in the range of 6.6–7.4, **(D)** NH_4_Cl concentrations in the range of 0.5–10 g/L, **(E)** glucose concentrations in the range of 0.5–20 g/L, and **(F)** medium volume (30, 50, 75, and 100 mL). All experiments were performed in 250 mL shake flask. Asterisks indicate significant difference (*p < 0.05, **p < 0.01, ***p < 0.001, *n* = 3).

To obtain the best fermentation parameters, we studied the growth curve of the rhaFGF_135_ engineered strain with culture time at different conditions. The engineered strain of rhaFGF_135_ presented a S-shaped growth curve, which showed the stagnation stage (0–2 h), logarithmic growth stage (3–9 h), and decline stage (after 12 h) when cultured at 37°C and 200 rpm (Figure 2A). The best parameters of temperature, pH, and concentrations of glucose and NH_4_Cl for the growth of rhaFGF_135_ strain were 37°C, 7.0, 5, and 4 g/L, respectively (Figures 2B,E). Effect of dissolved oxygen (DO) on the growth of rhaFGF strain was simulated with different volumes of LB medium. As shown in Figure 2F, 30 mL LB medium, which was equivalent to 30% dissolved oxygen, provided a better environment for *E. coli* growth.

**FIGURE 3:**
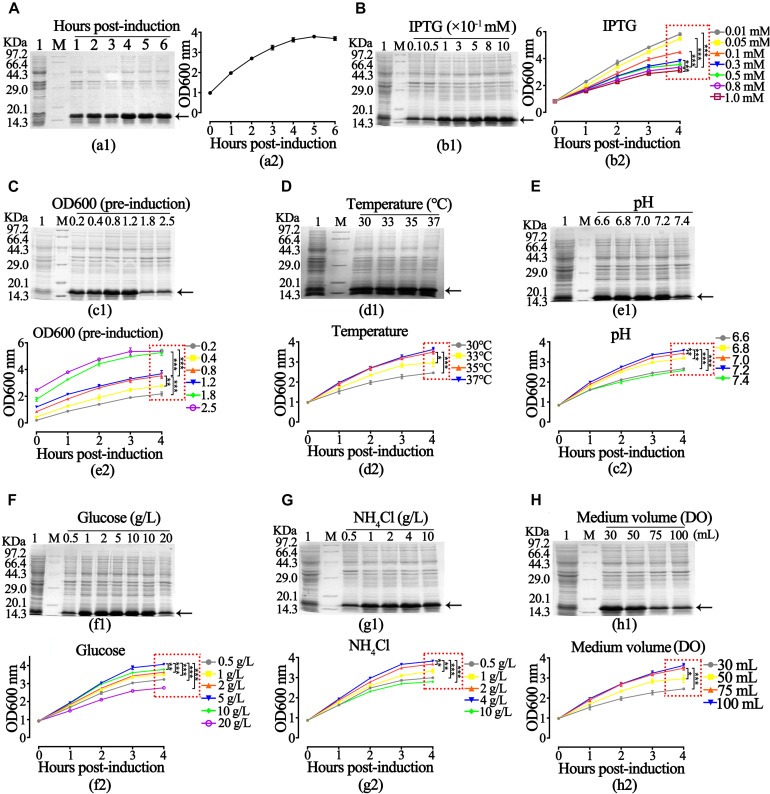
Optimization of expression parameters of rhaFGF_135_ *E. coli* strain in 30 mL LB medium. **(A)** The expression level of rhaFGF_135_ (a1) and bacterial density represented by OD_600_ (a2) induced by 0.8 mM IPTG for 1–6 h. **(B)** The expression level of rhaFGF_135_ (b1) and bacterial density (b2) after induction with 0.01–1.0 mM IPTG for 1–4 h. **(C–H)** The expression level of rhaFGF_135_ and bacterial density induced by 0.8 mM IPTG for 1–4 h under different conditions, including **(C)** pre-induction bacterial density (OD_600_), **(D)** temperature at 30, 33, 35, and 37°C, **(E)** pH in the range of 6.6–7.4, **(F)** glucose concentrations in the range of 0.5–20 g/L, **(G)** NH_4_Cl concentrations in the range of 0.5–10 g/L, and **(H)** medium volume (30, 50, 75, and 100 mL). Lane 1, non-induced. Lane M, molecular weight marker. All of the experiments were performed in 250 mL shake flask. Black arrows indicate rhaFGF_135_ protein. Asterisks indicate significant differences (*p < 0.05, **p < 0.01, ***p < 0.001, *n* = 3).

After obtaining the growth characteristics of rhaFGF strains, we studied the best expression parameters of rhaFGF_135_ in 250 mL shake flasks. As shown in Figures 3A–C, it was most appropriate to induce the protein expression at mid-logarithmic phase (OD_600_ = 0.8–1.2) with 0.8 mM IPTG for 4 h post-induction incubation. Then, the maximum production of rhaFGF_135_ was achieved after induction at 37°C and pH 7.0–7.2 (Figures 3D,E). Moreover, the optimum glucose concentration and NH_4_Cl concentration for induction were 5 and 4 g/L, respectively, which was consistent with those for cell growth (Figures 3F,G). Similarly, as shown in Figure 3F, high production of rhaFGF_135_ was achieved in 250 mL shake flasks with 30 mL LB medium, which the dissolved oxygen was also no less than 30%.

Subsequently, a relatively satisfactory result was derived from the preliminary evaluation of these optimized conditions at the 2-L scale fermentation (Supplementary Figure S1, Supplementary Table S1).

### Large-Scale Fermentation of rhaFGF_135_

**FIGURE 4:**
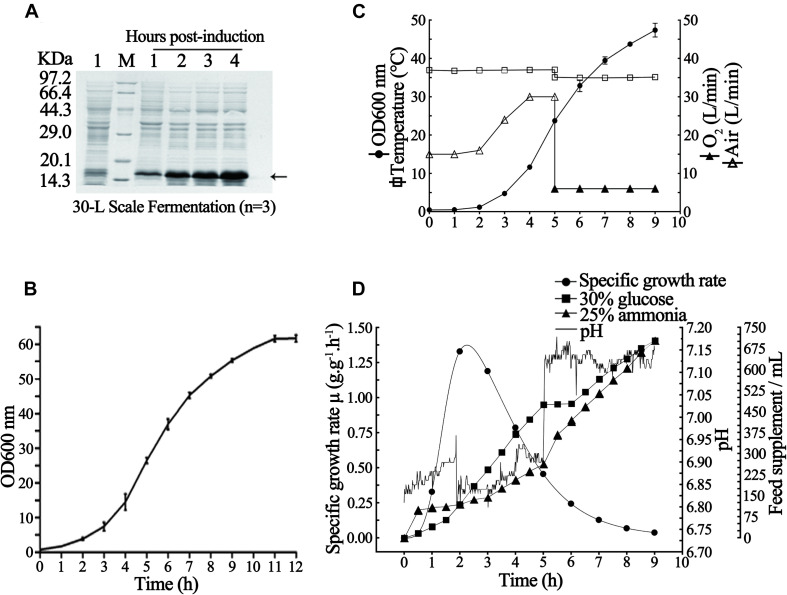
Fermentation process of *E. coli* BL21(DE3) plysS-pET3c/rhaFGF_135_. **(A)** rhaFGF_135_ expression SDS-PAGE analysis at the 30-L fermentation scale. Lane 1, non-induced. Lane M, molecular weight marker. **(B)** Growth curve of rhaFGF_135_ *E. coli* strain in a 30-L fermenter. **(C)** Variations in the parameters in the rhaFGF_135_ production at 30-L scale fermentation, including wet cell concentration (OD_600_), temperature, and ventilation rate of air or pure oxygen. **(D)** The relation curve of specific growth rate, glucose addition, pH, and ammonia addition with time in 30-L scale fermentation process. The arrow indicates rhaFGF_135_.

As shown in Figure 4B, the recombinant rhaFGF_135_ *E. coli* strain also presented an S-type growth curve in a 30-L fermenter at the abovementioned optimal growth conditions and reached the mid-logarithmic phase after incubation for 5 h. Subsequently, to establish the optimal conditions for improving production of rhaFGF_135_ at the 30-L scale fermentation, the above-mentioned fermentation parameters were slightly modified, and then three batches of fermentation were performed. During the cultivation process, the cell growth temperature was 37°C and the pH was maintained at 6.8–7.0 by adding 25% (v/v) ammonia solution using an automatic pH controller (Figures 4C,D); the DO was kept above 30% by gradually increasing the agitation speed from 200 to 650 rpm and augmenting the ventilation rate of air from 15 to 30 L/min (Figures 4C,D). After 5-hour culture, IPTG was added to the fermentation medium at a concentration of 0.8 mM when the culture reached the mid-logarithmic phase (OD_600_, 22–25) (Figure 4C). The pure oxygen (O_2_) was supplied at a rate of 6 L/min to keep the DO above 30% (Figure 4C). During the period of induction, the temperature and pH were stabilized at 35°C and 7.0–7.2, respectively (Figure 4C). Next, 1 h after the induction, the nitrogen source was added. Subsequently, 4 h after the induction, the bacterial wet weight and expression level of rhaFGF_135_ reached 80.4 ± 2.7 g/L culture and 37.8 ± 1.8%, respectively (Table 2, Figure 4A). Figures 4C,D depict various parameters implicated in the production of rhaFGF_135_ at a 30-L scale.

**TABLE 2:** Summary of fermentation data of rhaFGF_135_ at the scales of 30-L tank (*n* = 3).

Expression level (%)	Volume of fermentation liquid (L)	Bacterial density (g/L)	Bacterial wet weight (g)
37.8 ± 1.8	15.4 ± 0.2	80.4 ± 2.7	1241 ± 53

### Purification and Identification of rhaFGF_135_

**FIGURE 5:**
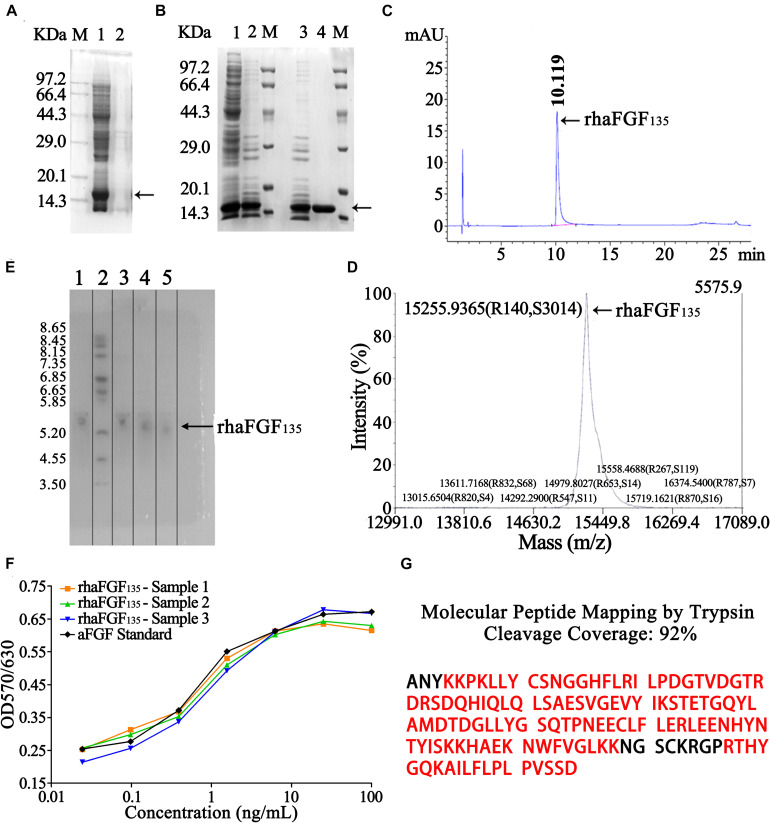
Analysis and identification of rhaFGF_135_ in the process of purification. **(A)** SDS-PAGE analysis of rhaFGF_135_ in bacteria lysis stage. Lane M, molecular weight marker. Lane 1, supernatant containing rhaFGF_135_. Lane 2, precipitate. **(B)** SDS-PAGE analysis of rhaFGF_135_ in ion exchange and affinity chromatography. Lane 1, supernatant. Lane 2 and 3, eluted sample with 0.6 M NaCl solution by CM-Sepharose. Lane 4, purified rhaFGF_135_ eluted with 1.3 M NaCl by heparin affinity-Sepharose. Lane M, molecular weight marker. **(C)** RP-HPLC analysis of purified rhaFGF_135_. **(D)** Mass spectrum analysis of purified rhaFGF_135_. **(E)** Isoelectric point analysis of purified rhaFGF_135_. Lane 1, rhaFGF standards. Lane 2, isoelectric point marker. Lanes 3–5, purified rhaFGF_135_ from three batches. **(F)** The biological activity of rhaFGF_135_ on NIH-3T3 cells. **(G)** Molecular peptide mapping coverage of rhaFGF_135_. The matched amino acids were marked in red. Black arrows indicate rhaFGF_135_ protein.

As shown in Figure 5A, the rhaFGF_135_ protein was expressed in a soluble state. Therefore, the stored frozen cell pellets (202.2 ± 1.9 g) were lysed (Table 3), and the supernatant was collected for further purification. Subsequently, the rhaFGF_135_ protein was purified by a combination of CM-Sepharose and heparin-affinity column chromatography, and the recovery of each step was 10.9% ± 1.8% and 36.5% ± 0.8% (Table 3). Detected by SDS-PAGE, the purity of rhaFGF_135_ obtained from each step was 43.3% ± 0.2% and 99.5% ± 0.1% (Table 3, Figure 5B). The summary of each purification step is presented in Table 3, and the final yield of purified rhaFGF_135_ was 158.6 ± 6.8 mg/L culture. As shown in Figure 5C, a single peak was present at 10.119 min after RP-HPLC analysis, which indicated that 100% purity of rhaFGF_135_ was achieved. Additionally, the authenticity of rhFGF_135_ was confirmed by MALDI-TOF/MS (Figure 5D), isoelectric point (Figure 5E), and western blotting (Supplementary Figure S2). As expected, the molecular weight and main isoelectric band of purified rhaFGF_135_ were 15,255.9365 Da (Figure 5D) and about 5.3 (Figure 5E), respectively. The *N*-terminal sequencing and molecular peptide mapping analysis were conducted by Shanghai Applied Protein Technology Co., Ltd., and the final 15 amino acid sequence of purified rhaFGF_135_ was ANYKKPKLLYCSNGG (data not shown), which matched with that in the NCBI database. As shown in Figure 5G, the molecular peptide mapping coverage of rhaFGF_135_ was 92% after cleavage by trypsin. The biological activity of the rhaFGF_135_ stock solution reached 9.2 ± 0.8 × 10^5^ AU/mg, which was similar to rhaFGF standard (Figure 5F).

**TABLE 3:** Summary of the purification steps of rhaFGF_135_ (*n* = 3).

Purification steps	Purification volume (mL)	Total protein (mg)	Target protein (mg)	Purity (%)	Recovery (%)
Bacteria lysis	2096.7 ± 11.5 (202.2 ± 1.9 g)^a^	10312.6 ± 1760.2	/	/	/
CM-Sepharose	428.3 ± 7.6	1099.2 ± 16.5	475.6 ± 8.4	43.3 ± 0.2	10.9 ± 1.8
Heparin affinity	208.3 ± 17.6	401.1 ± 3.5	399.1 ± 3.7^b^	99.5 ± 0.1	36.5 ± 0.8
Protein yield (mg/1 L culture)^c^: 158.6 ± 6.8					

*^a^The bacterial wet weight for purification. ^b^N = (P × W/W1)/S ≈ 24,000 bottles. ^c^Protein yield = (P × W/W1)/V (P: the amount of target protein obtained by heparin-affinity chromatography; W: the total bacterial wet weight; W1: the bacterial wet weight for purification; S: specification of Aifujifu, 100 μg/bottle; V: the volume of fermentation broth).*

### Efficiency and Pathological Evaluation of rhaFGF_135_ for Wound Healing

**FIGURE 6:**
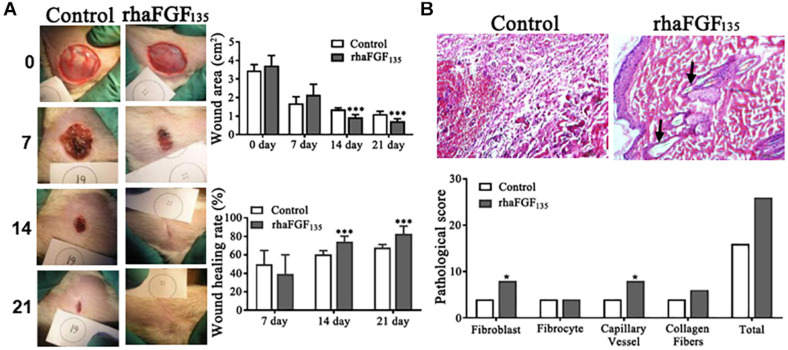
The healing effect of rhaFGF_135_ on full-thickness injury model in rats. **(A)** Photographs of skin wound and wound healing rates of STZ-induced SD rats with full-thickness wound. **(B)** Results of H&E staining (scale bar, 100 μm) and its pathological score in full-thickness injury model in rats. Black arrows indicate regenerated hair follicle. Compared with the control group, *p < 0.05, ***p < 0.001, *n* = 7.

Neither of the rats showed signs of infection during this experiment (Figure 6A). Compared with the control group, the rats treated with rhaFGF_135_ showed more significant shrinkage. The wound surface area on day 21 was 1.11 ± 0.14 cm^2^ in the control group and 0.73 ± 0.12 cm^2^ in the rhaFGF_135_ group (*p* < 0.001). The healing rate on day 21 was 68.1% ± 3.15% in the control group and 82.8% ± 8.32% in the rhaFGF_135_ group (Figure 6A).

After treatment for 21 days, three rats per group were euthanized, and the proliferation of fibroblasts, capillaries, and collagen fibers in the healing skin was observed via H&E staining. As showed in Figure 6B, the proliferation index of fibroblasts and neovasclarization were significantly increased in rhaFGF_135_ treatment group, while the fibrocyte and collagen fibers scores were increased, but without significant difference. Therefore, all of these data suggested that rhaFGF_135_ had a significant efficiency in promoting wound healing and improving microstructure of wounds in type 1 diabetic rats.

## Discussion

Human aFGF is a multifunction factor involved in a variety of biological processes, including angiogenesis, cell proliferation, and differentiation. To reduce the potential side effects or improve the stability of haFGF, several shortened or modified versions were developed, including haFGF_127_ (Lozano et al., 2000), haFGF_135_ (Cheng and Kuo, 2011), and TAT-haFGF_19__–__154_-His (Huang et al., 2008). As shown in Table 4, Wu et al. (2005) and Huang et al. (2008) reported the production of haFGF_127_ and TAT-haFGF_19__–__154_-His, respectively, in *E. coli* at the scale of 3-L fermentation, while Wang S. M. et al. (2009) have succeeded in expressing haFGF in *E. coli* at the 20-L fermentation scale. Yang et al. (2015) expressed the rhaFGF_135_ protein in *Arabidopsis thaliana* seeds by oleosin fusion technology. However, there have been practically no available reports or detailed studies regarding the larger scale fermentation and purification of rhaFGF_135_ in *E. coli*. In this study, we developed a stable and efficient process of 30-L scale high cell-density fermentation and purification protocol to achieve high production of the high-purity rhaFGF_135_ protein in *E. coli*.

**TABLE 4:** Comparison of total productivity of rhaFGF in different hosts.

Host	Scale of fermentation	Yield	Expression level	References
*Escherichia coli* MM294^a^	1 L	33.4 mg/L	/	Watanabe et al. (1990)
*Escherichia coli* AB1899^a^	2 L	3.6 mg/L	/	Zazo et al. (1992)
*Escherichia coli* BL(DE3)^b^	5 L	60 mg/L	25.0%	Wu et al. (2005)
*Escherichia coli* BL(DE3)^c^	5 L	171 mg/mL	36.7%	Huang et al. (2008)
*Escherichia coli* W3110^d^	20 L	/	29.11%	Wang S. M. et al. (2009)
*Pichia pastoris* ^a^	3 L	108 mg/L	/	Fantoni et al. (2007)
Silkworm^d^	/	2.6 mg/cocoon	26.0%	Wang et al. (2015)
*Nicotiana benthamiana* ^a^	/	10 μg/g leaf	/	Ha et al. (2017)
*Salvia miltiorrhiza* ^d^	/	272 ng/fresh leaf	/	Tan et al. (2014)
*Escherichia coli* BL(DE3)plysS	30L	158.6 mg/L	37.8%	Present study

*^a^Full length haFGF. ^b^haFGF_127_. ^c^TAT-haFGF_19__–__154_-His. ^d^haFGF_141_.*

*Escherichia coli* BL21(DE3) plysS was chosen as the host bacteria because it contains the small plasmid that encodes T7 lysozyme, which effectively inhibits the expression of non-target proteins without influencing the level of the target protein. Compared with *E. coli* JM109, *E. coli* BL21(DE3) accumulates much less acetic acid, and its cell culture density is higher by 25%. To enhance the specific synthesis rate of the target protein, we used 10% inoculation quantity of strains to shorten the lag phase during which the specific growth rate increases rapidly (within 1 h, as shown in Figure 4D). For example, Hu et al. (2009) were able to improve the final bacterial density and yield of HT-1 fusion protein (from 44.2 to 53.9 g/L and from 2.45 to 3.05 g/L) by increasing the inoculation quantity of strains from 5 to 10%.

Optimizing the fermentation parameters such as temperature, pH, and DO would create the most appropriate environment to obtain high yield of the rhaFGF_135_ protein in the recombinant *E. coli* strain. Glucose is a common carbon source for *E. coli* high-density fermentation. As shown in Figure 2E, the media containing 5.0–10 g/L glucose allowed *E. coli* cells to exhibit a better growth rate. When glucose concentrations were below 2.0 g/L, the growth of cells was inhibited due to the deficiency of energy. However, when glucose concentrations exceeded 10 g/L, it significantly suppressed cell growth due to the production of large amounts of acetic acid. Interestingly, as shown in Figure 3F, after induction, both the expression level of rhaFGF_135_ and the culture density (OD_600_) were relatively higher in the medium containing 5 g/L glucose. Therefore, in this study, during 30-L scale fermentation, the feedback parameter pH was taken as the control, which was associated with the additional supplement of glucose to adjust the glucose concentration and specific growth rate, so as to control the production of acetic acid and improve the expression of rhaFGF_135_ (Figure4D). As shown in Figure 4D, within the first 3 h of fermentation, along with glucose addition, the specific growth rate of bacteria increased; thereafter, during the period of induction (after 5 h of fermentation), the specific growth rate of bacteria decreased with the decrease of the glucose addition rate.

PH is another key parameter with a great effect on bacterial growth and target protein expression. Recently, it has been reported that the optimal pH for cell growth and target protein accumulation differs depending on the stage of culture (Wang J. et al., 2009). Herein, we found that the optimal pH for the growth of recombinant *E. coli* strain and the expression of rhaFGF_135_ was 6.8–7.0 (Figure 2C) and 7.0–7.2 (Figure 3E), respectively. In this study, during 30-L scale fermentation, we used 25% ammonia solution to neutralize the excess acid in the culture medium and glucose solution to neutralize alkaline substances by producing the metabolites of acetic acid. As shown in Figure 4D, the incessant proliferation of cells in the growth stage was accompanied by increasing glucose consumption, which led to higher concentrations of acid species in the medium. These species were neutralized by gradually increasing the amount of 25% ammonia solution added to the culture. During the expression stage, the rate of glucose addition was gradually decreased, while the rate of ammonia addition remained almost constant (Figure 4D), which indicated that the production rate of acetic acid was almost unchanged. Moreover, it should be noted that in addition to its neutralization effect, 25% ammonia solution could be also used as a nitrogen source in the medium.

Temperature constitutes another important parameter of high-density fermentation. Liu et al. (1999) suggested that higher temperatures are more beneficial for bacterial growth, whereas lower temperatures can improve the yield of recombinant products. Herein, 37°C was the optimal growth temperature of the recombinant rhaFGF_135_ strain, considering that it presented the highest cell density (OD_600_) at all growth stages (Figure 2B). The expression level of the protein was similar in the range of 30–37°C; however, the cell density (OD_600_) was higher at 35 and 37°C (Figure 3D). Considering the energy consumption of fermentation, the optimal induction temperature of the 30-L scale process was set at 35°C (Figure 4C).

The oxygen dissolved in the culture medium is involved in all stages of high-density fermentation, especially the middle and late stages. In fact, oxygen deficiency enhances the production of acetic acid, thereby inhibiting protein synthesis and bacterial growth. Moreover, excessively high or low dissolved oxygen contents are detrimental to the formation of recombinant products. As shown in Figures 2F and 3H, dissolved oxygen level >30% favored the growth of the rhaFGF_135_ engineering strain and the highest expression of this protein. To maintain a constant level of dissolved oxygen during the fermentation process, the agitation speed and ventilation rate were gradually increased, and pure oxygen gas was added to the culture medium (after 5 h of fermentation).

IPTG, a compound commonly used to induce the expression of recombinant products in *E. coli*, was chosen as an inducing agent. Previously, 1 mM IPTG was used to induce rhaFGF and rhKGF-2 (Wu et al., 2005, 2009). In this study, as shown in Figure 3B, the expression level of rhaFGF_135_ increased with the increase of IPTG concentration (0.01 to 0.5 mM), while it was similar in the range between 0.5 and 1.0 mM IPTG. However, the cell growth was gradually suppressed with the increase of IPTG concentration (0.01 to 1.0 mM). Combined with previous experience, 0.8 mM IPTG was then applied for 30-L scale fermentation. Consistent with other studies, the maximum expression of the rhaFGF_135_ protein could be attained after 4 h of induction, when the culture reached the mid-logarithmic phase (Figures 3A,C).

Microelements and inorganic ions, such as Ca^2+^, Mg^2+^, Na^+^, NH^4+^, K^+^, and vitamin B1, are typically used to support the normal growth and metabolism of bacteria. Previously, Reiling et al. (1985) proposed that the basic fermentation medium of *E. coli* should contain 0.77 g/L NH_4_Cl, 0.125 g/L KH_2_PO_4_, 0.4 mg/L CaCl_2_, 17.5 mg/L MgSO_4_.7H_2_O, 7.5 mg/L KH_2_PO_4_.7H_2_O, and 0.64 mg/L FeSO_4_.7H_2_O. Xu et al. (1998) reported that the final bacterial density and expression level of the target protein were most significantly affected by the phosphate (PO_4_^3–^) content of the medium, which might be due to the effect of phosphate in changing the replication rate of the plasmid in *E. coli*. Zhang et al. (2006) indicated that the addition of Mg^2+^ at a concentration of 1 g/L could significantly improve the growth of recombinant *E. coli* BL21 (DE3) and the expression level and stability of the target protein. It could also enhance the stability of the plasmid by decreasing its loss rate from 15.4 to 1.1% and inhibit the autolysis of bacteria (Zhang et al., 2006). As a cofactor involved in the oxidative decarboxylation of pyruvate and α-ketoglutarate, vitamin B1 (VB1) plays an important role in the growth of recombinant *E. coli* and production of target protein (Guo et al., 2014; Yuan et al., 2015). Herein, we used 4 g/L NH_4_Cl as the optimum concentration to promote the proliferation of *E. coli* during fermentation (Figures 2D, 3G). Moreover, 4.0 g/L NaCl, 20 mM PO_4_^3–^, 1.0 g/L MgSO_4_.7H_2_O, 13 mg/L CaCl_2_, and 5 mg/L VB1 to the 30-L were added to the fermentation medium to improve bacterial growth and protein expression. Overall, the medium cost is about $65.

Under the contribution of these optimized fermentation conditions, the yield (158.6 ± 6.8 mg/L culture) and expression level (37.8% ± 1.8%) of the rhaFGF_135_ protein were significantly improved, compared with prior studies (60 mg/L culture and 25%, respectively) (Wu et al., 2005). The protein expression level achieved herein (37.8% ± 1.8%) was also higher than those reported previously (29.11%, 36.7%) (Huang et al., 2008; Wang S. M. et al., 2009).

The rhaFGF_135_ protein was expressed mostly in soluble form and then purified by using a combination of CM-Sepharose and heparin-affinity column chromatography, which is consistent with the purification of haFGF_127_ (Wu et al., 2005). The following improvements were made. To protect the target protein and promote bacterial lysis, 0.05% Tween-80 was added to the extraction solution before purification. The buffer solutions of column chromatography were also supplemented with EDTA-2Na (5 mmol/L) in order to reduce the oxidation of metal ions in the target protein. Furthermore, the pH value of these buffer solutions was adjusted from 7.4 to 7.0, to diminish the negative charge of rhaFGF_135_ and enhance its binding capacity to the CM-Sepharose column. Finally, for heparin-affinity column chromatography, the concentration of NaCl in the elution buffer was increased from 1.2 to 1.3 mol/L to augment the final protein yield. Consequently, the recovery of CM-Sepharose and heparin-affinity column chromatography reached 10.9 and 36.5%, respectively (Table 3), which was higher compared with a previous study (about 3.6 and 22.3%, respectively) (Wu et al., 2005). Ultimately, these improvements also increased the purity of the eluted protein from 95 to 100% and the protein yield from 60 to 158.6 ± 6.8 mg/L culture (Tables 3, 4). Moreover, as shown in Table 4, compared with the expression of haFGF in other hosts, the yield of rhaFGF_135_ achieved herein was quite satisfying. As shown in Table 3, we also estimated that the total purified rhaFGF_135_ protein obtained from a 30-L scale fermentation could produce about 24,000 bottles of Aifujifu, which is a marketed drug of rhaFGF (100 μg/bottle). It indicates that the purification protocol herein may set the foundation for rhaFGF_135_ purification at the industrial level.

Moreover, accumulating evidence indicates that the localization sequence from *N*-terminal at position 21 to 27: NYKKPKL is indispensable for the mitogenic activity of aFGF (Imamura et al., 1990, 1992; Lozano et al., 2000). In the MTT assay, rhaFGF_135_ showed a comparable mitogenic activity to the haFGF standard (Figure 5F), which was consistent with the previous studies (Wu et al., 2004; Huang et al., 2008). Moreover, in the clinical setting, aFGF is mainly applied for wound healing; for example, Aifujifu, a native mature rhaFGF consisting of 141 amino acids, was approved by China Food and Drug Administration (CFDA) for treating scalding injuries and chronic ulcers (Sun et al., 2015). As shown in Figure 6, rhaFGF_135_ significantly promoted wound healing and improved microstructure of wounds in type 1 diabetic rats. Therefore, our data indicated that rhaFGF_135_ may have a very similar mitogenic activity to haFGF_141_ or haFGF_154_ *in vivo* and *in vitro*.

## Conclusion

In conclusion, we successfully constructed an *E. coli* BL21(DE3)plysS-pET3C/rhaFGF_135_ engineering strain and established a stable and efficient high-cell-density fermentation process at the scale of 30-L. A purification protocol of the rhaFGF_135_ protein produced on a large scale was also developed. The methods proposed herein set the foundation for rhaFGF_135_ production at the industrial level.

Abbreviations: bFGF, basic fibroblast growth factor; CFDA, China Food and Drug Administration; DMEM, Dulbecco’s modified eagle medium; DMSO, dimethyl sulfoxide; DO, dissolved oxygen; FBS, fetal bovine serum; H&E, hematoxylin and eosin staining; IL-1, interleukin-1; IPTG, isopropyl-β-D-thiogalactoside; MTT, 3-(4,5-dimethyl-2-thiazolyl)-2,5-diphenyl-2-H-tetrazolium bromide; OD_600_, optical density at 600 nm; PB, phosphate buffer; PCR, polymerase chain reaction; rh-aFGF_135_, recombinant human acid fibroblast growth factor_135_; RP-HPLC, reverse-phase high-performance liquid chromatography; SDS-PAGE, sodium dodecyl sulfate polyacrylamide gel electrophoresis; STZ, streptozocin.

## Data Availability Statement

The datasets presented in this study can be found in online repositories. The names of the repository/repositories and accession number(s) can be found below: https://www.ncbi.nlm.nih.gov/genbank/, MT150274.

## Ethics Statement

The animal study was reviewed and approved by Wenzhou Medical University (Zhengjiang, China).

## Author Contributions

BY, WS, ZH, GS, LL, JG, and MZ performed the experiments. QH, XL, and CC contributed to experiments. ZH and GS contributed to data analysis. XW and QH designed, supervised, and coordinated the study. BY wrote the manuscript. WS contributed to manuscript editing. All of the authors read and approved the final manuscript.

## Conflict of Interest

The authors declare that the research was conducted in the absence of any commercial or financial relationships that could be construed as a potential conflict of interest.
