# Corrigendum: Large-scale preparation of highly stable recombinant human acidic fibroblast growth factor in *Escherichia coli* BL21(DE3) plysS strain

**DOI:** 10.3389/fbioe.2022.1048797

**Published:** 2022-10-10

**Authors:** Bingjie Yu, Wenzhe Sun, Zhen Huang, Gang Sun, Le Li, Jiawei Gu, Mengying Zheng, Xiaokun Li, ChangJu Chun, Qi Hui, Xiaojie Wang

**Affiliations:** ^1^ Wenzhou Medical University, Chashan University Park, Wenzhou, China; ^2^ College of Pharmacy, Chonnam National University, Gwangju, South Korea; ^3^ Engineering Laboratory of Zhejiang Province for Pharmaceutical Development of Growth Factors, Biomedical Collaborative Innovation Center of Wenzhou, Wenzhou, China; ^4^ Research Institute of Pharmaceutical Sciences, College of Pharmacy, Chonnam National University, Gwangju, South Korea

**Keywords:** rhaFGF_135_, *Escherichia coli* BL21(DE3)pLysS, fermentation, purification, wound healing

In the published article, an author name was incorrectly written as Bingjieu Yu. The correct spelling is “Bingjie Yu”.

Furthermore, there was an error regarding the affiliation for Bingjie Yu. Beyond having affiliation 1, they should also have affiliation 2 (added here), “College of Pharmacy, Chonnam National University, Gwangju, South Korea.”

The authors apologize for this error and state that this does not change the scientific conclusions of the article in any way. The original article has been updated.

