# Supplementary material for: Large-Scale Preparation of Highly Stable Recombinant Human Acidic Fibroblast Growth Factor in Escherichia coli BL21(DE3) plysS Strain
Source: Front Bioeng Biotechnol. 2021 Apr 13;9:641505. doi: 10.3389/fbioe.2021.641505 (PMC8072344; doi:10.3389/fbioe.2021.641505)
Supplement: Supplementary file 1 [file Presentation_1.PPTX]

## Slide 1
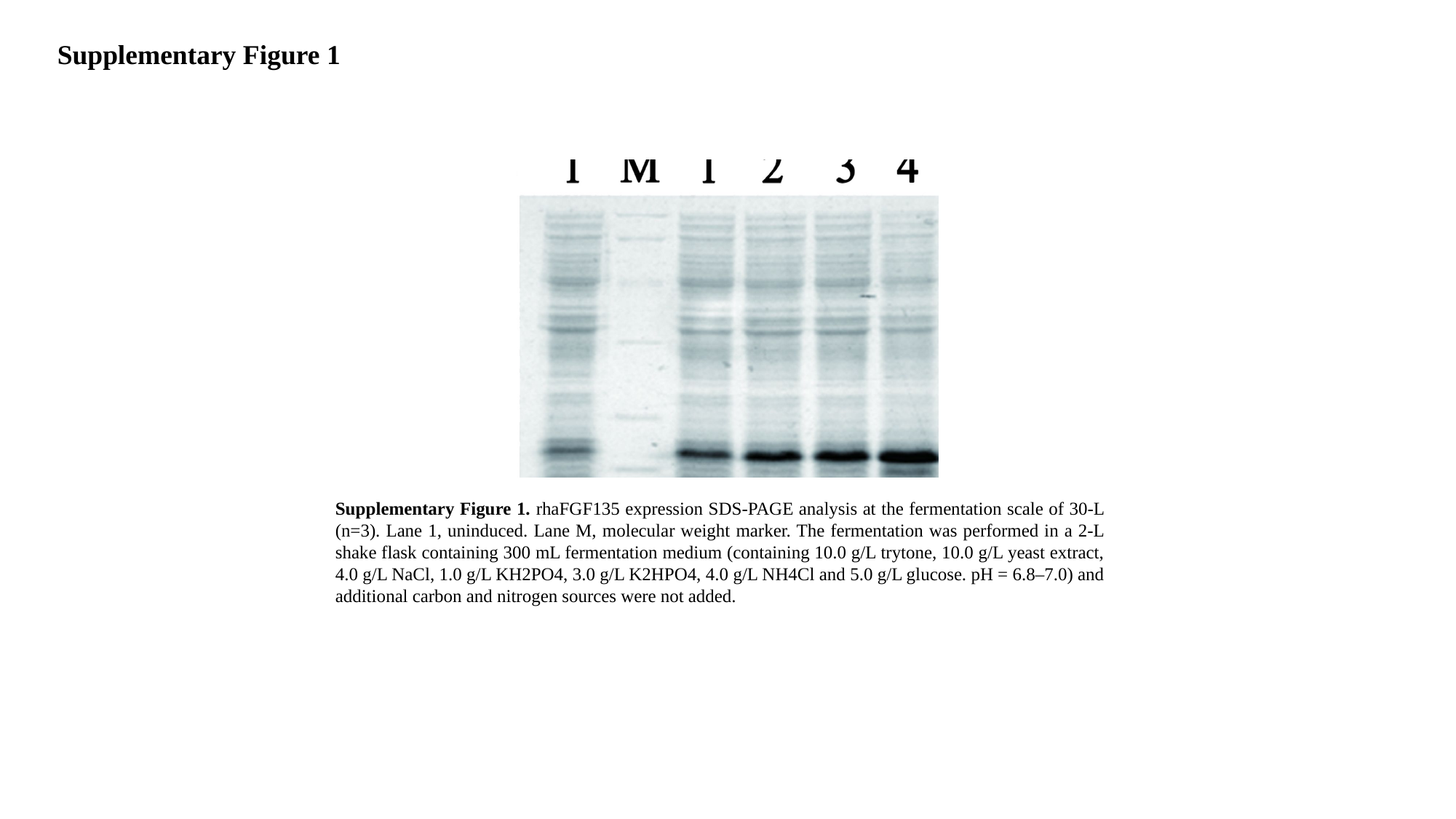

Supplementary Figure 1
Supplementary Figure 1. rhaFGF135 expression SDS-PAGE analysis at the fermentation scale of 30-L (n=3). Lane 1, uninduced. Lane M, molecular weight marker. The fermentation was performed in a 2-L shake flask containing 300 mL fermentation medium (containing 10.0 g/L trytone, 10.0 g/L yeast extract, 4.0 g/L NaCl, 1.0 g/L KH2PO4, 3.0 g/L K2HPO4, 4.0 g/L NH4Cl and 5.0 g/L glucose. pH = 6.8–7.0) and additional carbon and nitrogen sources were not added.

## Slide 2
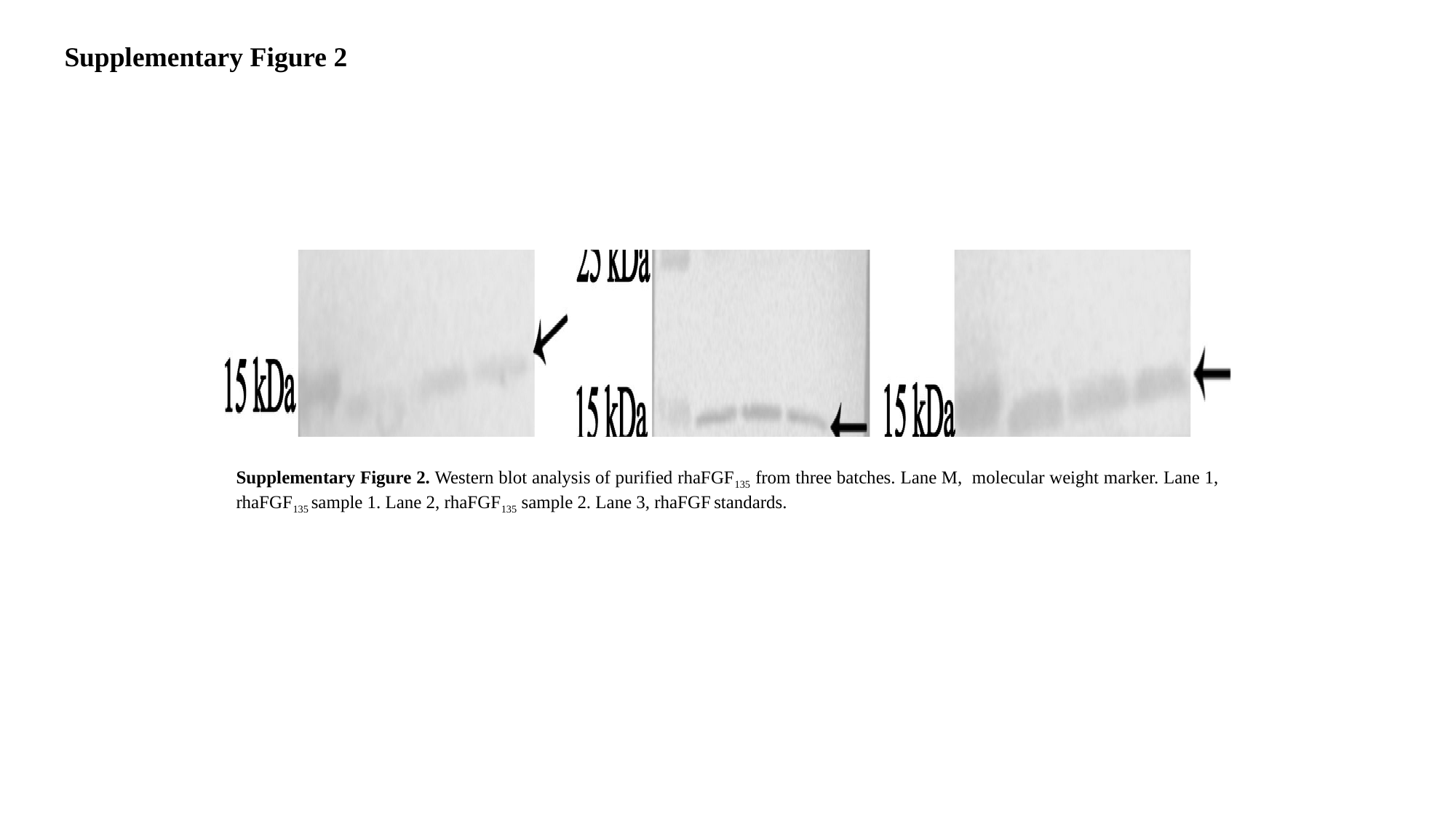

Supplementary Figure 2
Supplementary Figure 2. Western blot analysis of purified rhaFGF135 from three batches. Lane M, molecular weight marker. Lane 1, rhaFGF135 sample 1. Lane 2, rhaFGF135 sample 2. Lane 3, rhaFGF standards.

## Slide 3
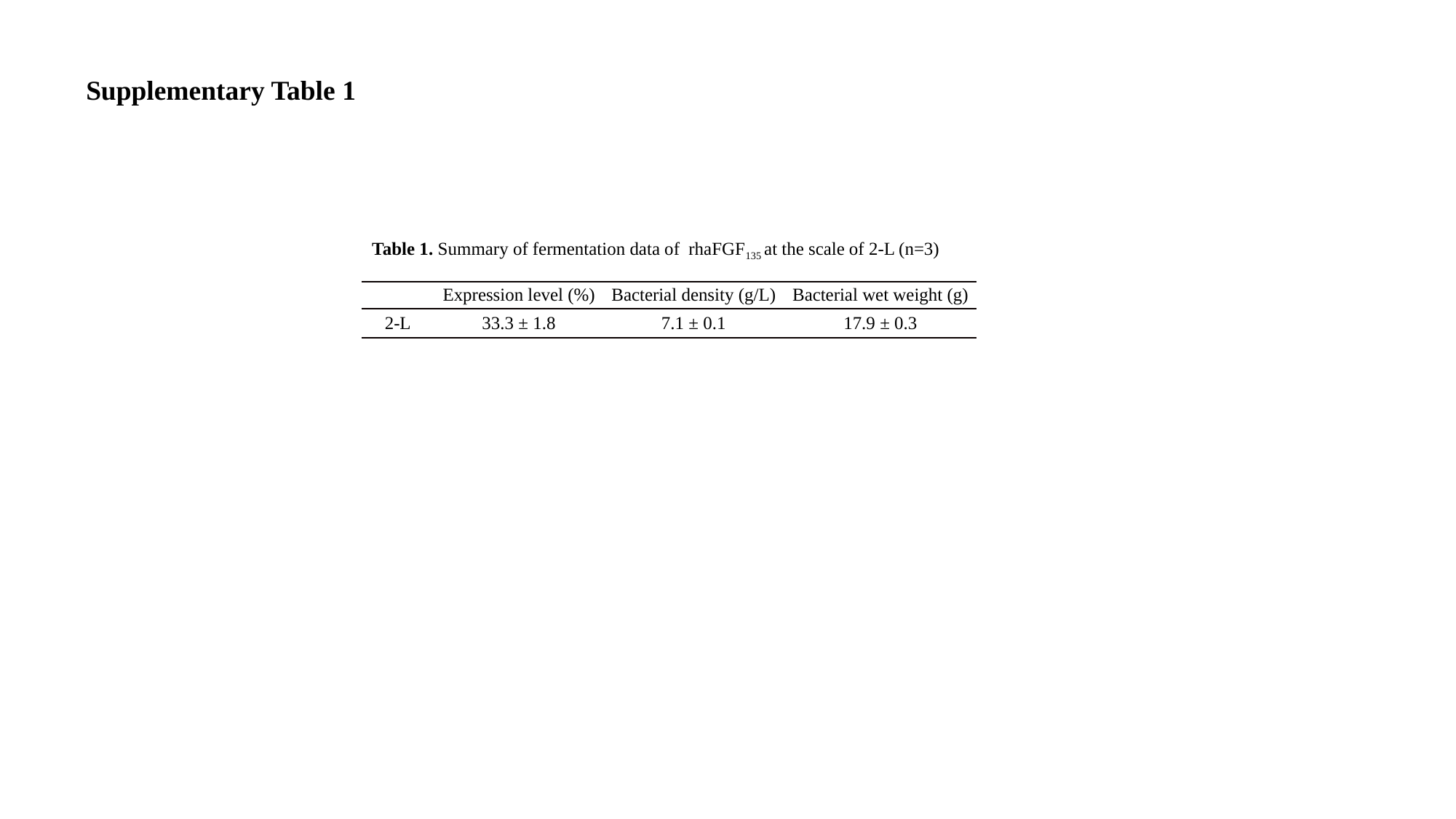

Supplementary Table 1
Table 1. Summary of fermentation data of rhaFGF135 at the scale of 2-L (n=3)
| | Expression level (%) | Bacterial density (g/L) | Bacterial wet weight (g) |
| --- | --- | --- | --- |
| 2-L | 33.3 ± 1.8 | 7.1 ± 0.1 | 17.9 ± 0.3 |
